# Resolving a conservation dilemma: Vulnerable lions eating endangered zebras

**DOI:** 10.1371/journal.pone.0201983

**Published:** 2018-08-29

**Authors:** Timothy G. O’Brien, Margaret F. Kinnaird, Steven Ekwanga, Christopher Wilmers, Terrie Williams, Alayne Oriol-Cotterill, Daniel Rubenstein, Laurence G. Frank

**Affiliations:** 1 Wildlife Conservation Society, Global Programs, Bronx, New York, United States of America; 2 Mpala Research Centre, Nanyuki, Kenya; 3 Living with Lions, Nanyuki, Kenya; 4 Center for Integrated Spatial Research Environmental Studies Department, University of California, Santa Cruz, California, United States of America; 5 Department of Ecology and Evolutionary Biology, Center for Ocean Health, University of California, Santa Cruz, California, United States of America; 6 Ewaso Lions, Nairobi, Kenya; 7 Wildlife Conservation Research Unit, Zoology Dept, University of Oxford, Tubney, United Kingdom; 8 Dept of Ecology and Evolutionary Biology, Princeton University, Princeton, New Jersey, United States of America; 9 Museum of Vertebrate Zoology, University of California, Berkeley, California, United States of America; Michigan Technological University, UNITED STATES

## Abstract

When predators are removed or suppressed for generations, prey populations tend to increase and when predators are re-introduced, prey densities should fall back to pre-control levels. In cases of apparent competition where there are alternate abundant and rare prey species, rare species may decline further than expected or disappear altogether. Recently, concern about the impact of recovering predator populations on wildlife in Laikipia County, Kenya, has led to questions of whether lions (*Panthera leo*, IUCN Red List Vulnerable) exert top-down pressure on Grevy’s zebra (*Equus grevyi*, IUCN Red List Endangered). We examined effects of lion predation on Plain’s zebra (*E*. *quagga*, IUCN Red List Near Threatened) and Grevy’s zebra populations in a 2,105 km^2^ area defined by lion movements. We used line transect surveys to estimate density of Grevy’s (0.71/km^2^) and Plain’s (15.9/km^2^) zebras, and satellite telemetry to measure movements for lions and both zebras. We tracked lions to potential feeding sites to estimate predation rates on zebras. We compared field-based estimates of predation rates on both zebras to random gas models of encounters that result in predation to ask if lions prey preferentially on Grevy’s zebra at a sufficient rate to drive population declines. Lions preyed on Grevy’s zebra significantly less than expected in 15 of 16 (94%) scenarios considered and lions preyed on Plain’s zebras as expected or significantly less than expected in 15 of 16 scenarios. Population trend of Grevy’s zebra indicates that the Kenya population may be stabilizing. Recruitment rate to the population has tripled since 2004, making it unlikely that lions are having an impact on Grevy’s zebras. In Laikipia County, competitive displacement by livestock (Livestock: Grevy’s zebra ratio = 864:1) and interference competition for grass with Plain’s zebra (Plain’s zebra:Grevy’s zebra ratio = 22:1) are most likely the predominant threats to Grevy’s Zebra recovery.

## Introduction

Large carnivores are known to influence the dynamics, distribution and behavior of ungulate prey populations [1, 2, 3]. Trophic cascades, top-down forcing effects of predators upon prey and thus on vegetation, are now widely recognized as major contributors to ecosystem structure and function [4]. Removal of predators can lead to high densities of prey and may allow them to concentrate on preferred food resources, leading to overutilization and degradation of vegetation on both local and landscape scales [1, 5, 6](but see [7]). Removal of large predators may also affect the behavior of prey populations. Over time, lack of predators may result in prey becoming unfamiliar with predator risk, making them more naïve and vulnerable to re-introduced or recovering predator populations. In ecosystems where humans have eliminated predators and then allowed or assisted predator recovery, we often see reduction in prey populations as predator and prey densities return to pre-removal levels. In some systems however, where communities of prey populations include very common and very rare species, apparent competition and opportunistic predation can lead to disproportionate reduction in the density of the rarer species [8, 9].

Restoration or maintenance of top predators may conflict with other conservation priorities when successful carnivore conservation in turn threatens a rare prey species. Policy makers and managers then need to address conflicting outcomes in endangered species interactions. In predator-prey relationships, conservation policy and management decisions should be based upon sound data and a thorough understanding of the predator-prey dynamics. In reality, such decisions often are complicated by sociopolitical considerations when predator, prey or both are endangered, protected, charismatic or otherwise in the public eye. For example, off the California coast, endemic Channel Islands foxes (*Urocyon littoralis*) declined after the islands were colonized by golden eagles (*Aquila chrysaetos*) in response to local extinction of native bald eagles (*Haliaeetus leucocephalus*) from DDT [10]. Foxes recovered rapidly following translocation of golden eagles from the islands. Although they are protected in California, mountain lions (*Puma concolor)* that prey on endangered Sierra bighorn sheep (*Ovis canadensis sierrae)* have been selectively removed as part of the Sierra Nevada Bighorn Sheep Recovery Program [11]. In other cases, predator removal is not an option: collapse of sea otter (*Enhydra lutris*) and Steller sea lion (*Eumetopias jubatus*) populations in the Aleutian Islands have been attributed to increased predation by killer whales *(Orcinus orca*), a result of prey-switching by killer whales after losing their former prey base of great whales to post World War II industrial whaling in the North Pacific [12, 13].

**Fig 1:**
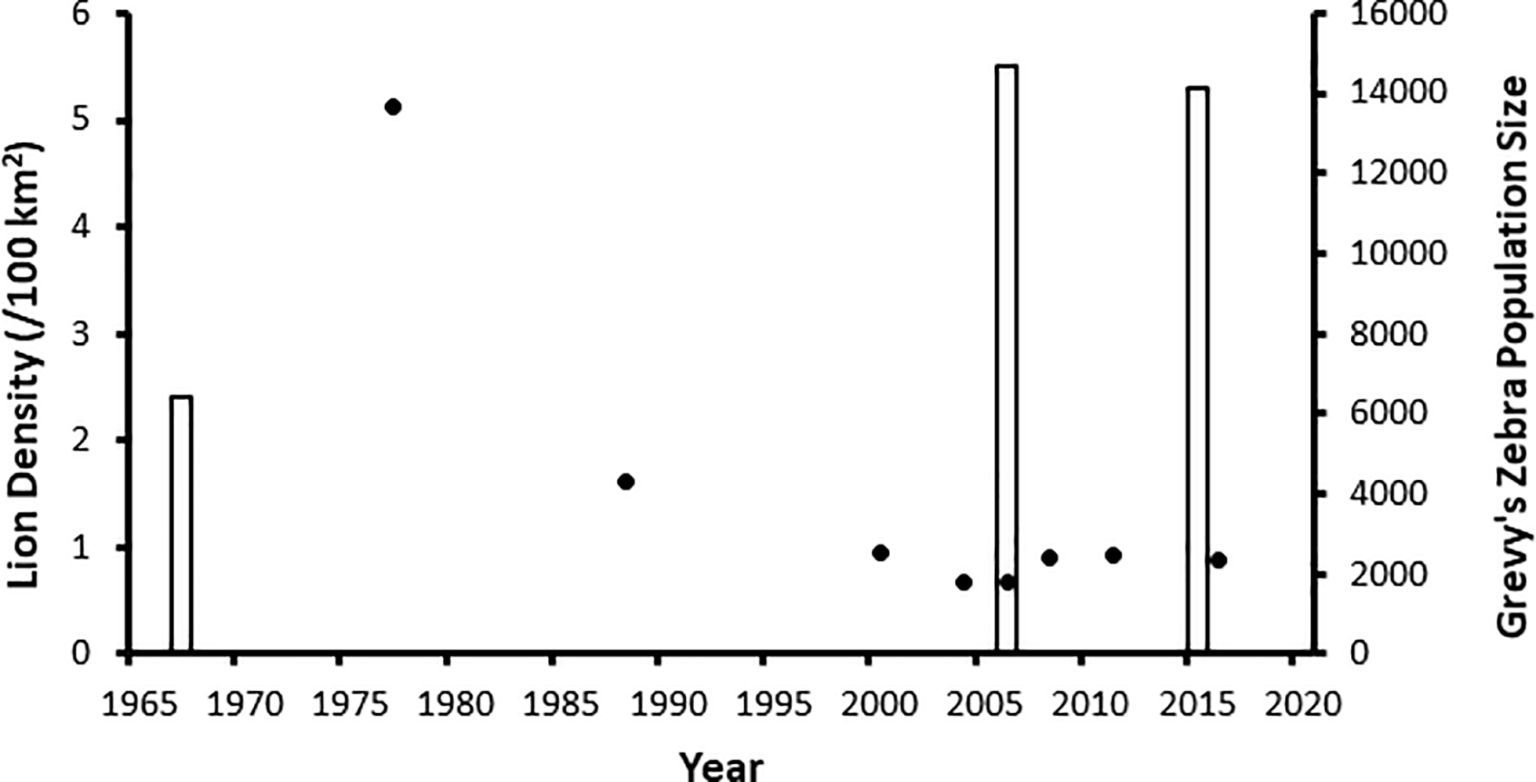
Population trends. Lion density estimates for Laikipia County (open bars) and estimates of Kenya’s Grevy’s zebra abundance (closed circles) between 1977 and 2016.

The Grevy’s zebra (*Equus grevyi*) is classified as Endangered on the IUCN Red List, due to a population decline throughout its range from an estimated 13,700 in 1977 to an estimated 2,680 individuals in 2016 throughout the range [14] (Fig 1). Today, Grevy’s zebra occurs primarily in Laikipia and Samburu Counties, Kenya, with small isolated populations in southern Ethiopia. Lions (*Panthera leo*), the primary natural predators of Grevy’s zebra, also have been in steep decline, extirpated from over 80% of their historical range and reduced to an estimated population of 20,000–30,000 across Africa [15]. Lions are classified as Vulnerable by the IUCN and the Kenya Wildlife Service and Threatened by the US Fish and Wildlife Service (http://ecos.fws.gov/).

Laikipia County, Kenya provides refuge for an estimated 1,400 (>50%) of the world’s remaining Grevy’s zebras. The county also has a stable lion population estimated at 5.3 adult and subault lions per 100 km^2^ [16, 17]. Historically, livestock production was the major land use in Laikipia County, and ranchers practiced predator control, primarily by shooting and poison [16] such that the lion population was suppressed, estimated at 2.4/100 km^2^ in the late 1960’s [18] (Fig 1). However, as livestock profits declined in the 1980’s and 1990’s, ranches turned to wildlife tourism and, as conservation became a high priority on most properties [19], predator tolerance increased and lion populations recovered. Today most ranchers shoot problem lions only as a last resort [16, 20, 21].

Since the late 1990’s, many large ungulate populations, including Grevy’s zebra, have been declining in Laikipia County [19, 22], whereas the Plains zebra populations has stayed relatively stable. Georgiadis et al. [23] speculated that Laikipia’s recovering predator community, combined with climate anomalies caused by ENSO-related cycles of droughts and heavy rains, were driving declines among large ungulate populations. They argued that, as the Laikipia landscape shifted from wildlife persecution to wildlife tolerance in the 1980’s and 1990’s, Plain’s zebra (*Equus quagga*) abundance increased 5-fold, providing an abundant food supply for recovering predator populations. Droughts caused widespread livestock and wildlife mortality that provided abundant carrion, and periods of high rainfall resulted in taller grasses and better cover for hunting predators. Although a century of fire suppression has caused large scale landscape transition from grassland to bush [24], Georgiadis et al. [23] surmised that declines in grassland ungulate species, such as Jackson’s hartebeest (*Alcephalus buselaphus*) and eland (*Tragelaphus oryx*), were due primarily to increasing predation pressure. A recent study by Ng’weno et al. [9] demonstrated that lion predation can drive the demography of Jackson’s hartebeest in Ol Pejeta, a 294 km^2^ fenced conservancy in Laikipia with a very high lion density (~24 lions/100 km^2^), and that increasing bush encroachment contributed to the decline.

Rubenstein [25] explicitly focused on the role of lions in limiting endangered Grevy’s zebra numbers. He based his assessment on observations from Lewa Wildlife Conservancy (LWC), a 250 km^2^ fenced rhinoceros reserve neighboring Laikipia County. In 2000, LWC introduced lions to a naïve community of large ungulates; within three years, population counts showed a 17% and 19% reduction in Plain’s and Grevy’s zebras, respectively (LWC, unpublished data). In an analysis of LWC lion scat, Rubenstein [25] found that, although Plain’s zebra were much more abundant than Grevy’s zebra, Grevy’s zebra hair was more common than Plain’s zebra hair and concluded that lions were preferentially selecting Grevy’s zebra over Plain’s zebra. Although Plain’s and Grevy’s zebra have continued to decline on LWC, it is unclear whether lions or other ecological pressures (e.g. fencing or competition for grazing from Plains zebra) drive these declines.

These observations have raised concerns about the impact of lion predation on the endangered Grevy’s zebra and the potential for contradictory conservation goals: successful lion conservation may impede successful Grevy’s zebra conservation in Laikipia County and elsewhere. The studies above, however, either were unable to establish a causal link [23] or were based on small data sets [25], leaving the question open as to whether lions are driving the population declines of Grevy’s zebra in Laikipia County, Kenya.

To answer this question, we evaluated lion predation on Grevy’s and Plain’s zebras across a large, mixed-use landscape in Laikipia County using satellite telemetry to track lions and zebra movements, and to locate and identify lion kills. We used random gas models to address the question of whether lions are preying on Grevy’s zebra more than expected due to chance alone and to assess the likelihood that lions are negatively influencing Grevy’s zebra numbers.

## Methods

### Study area

**Fig 2:**
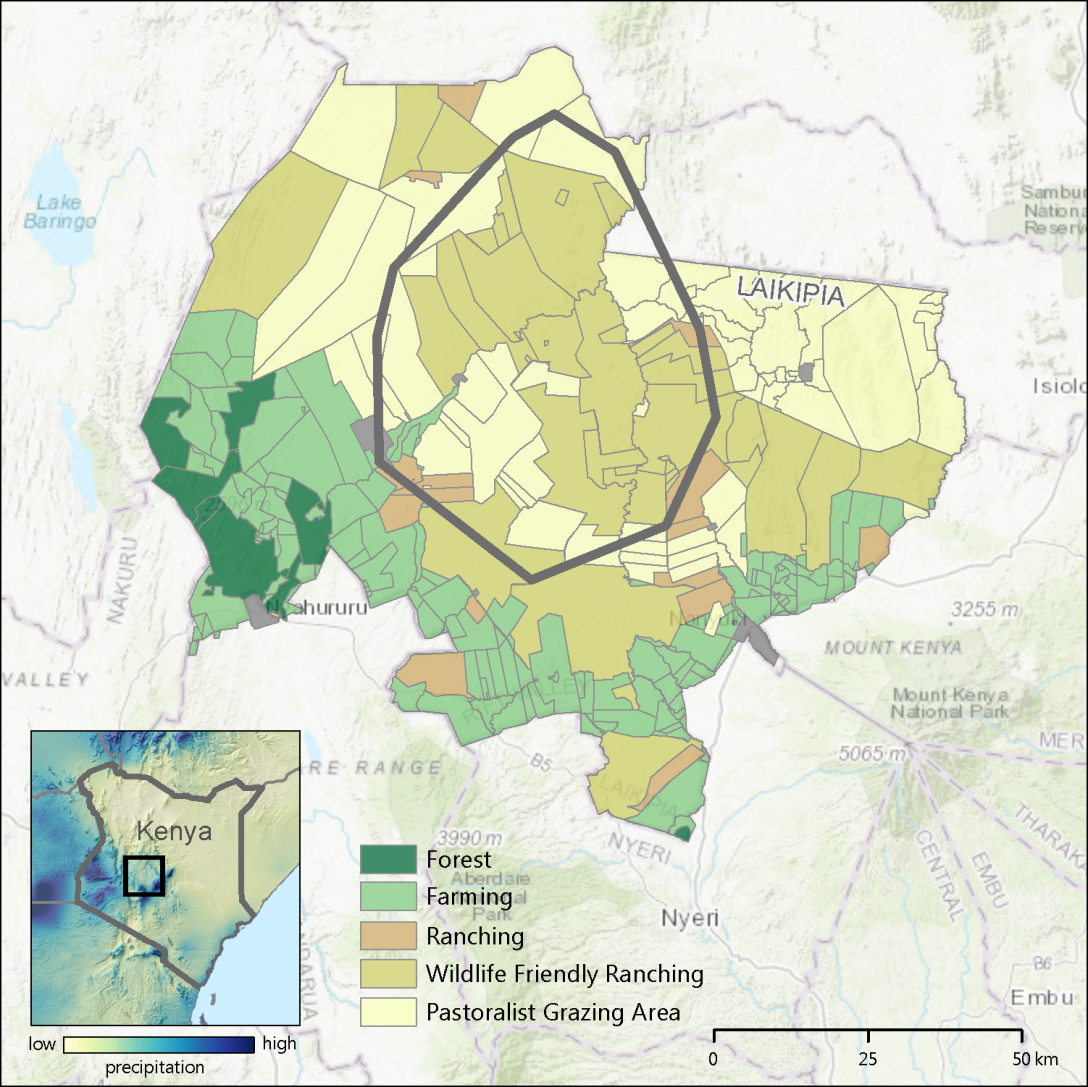
Study area. Location of study area within Laikipia County, Kenya. This figure was generated by the authors using lion telemetry data for minimum convex polygon, landuse shapefiles from the Mpala Research Centre open access GIS database, and ESRI ARCGIS. This figure is not copyrighted.

We conducted our study in Laikipia County, Kenya (9,666 km^2^: Fig 2). Laikipia is characterized by a mosaic of land uses and land management practices ranging from large, commercial cattle ranches and wildlife conservancies to smaller, densely populated pastoral group ranches and subsistence agricultural plots. Laikipia County is an excellent example of wildlife conservation on private lands and hosts the most diverse and second largest wildlife community in Kenya after Maasai Mara National Reserve [26].

Rainfall varies from 1000 mm at the foot of Mt. Kenya on the equator to 400 mm in the north of the county. Consequently, humans and permanent agriculture are concentrated in the south and wildlife conservancies, commercial ranches and group ranches (communally owned pastoralist properties) are concentrated in the north. Our study focused on a 2,770 km^2^ area in northern Laikipia (Fig 2), a largely unfenced area of ranches where populations of Plain’s zebra, Grevy’s zebra, many other wild ungulates, livestock, lions and other large carnivores co-occur. Three vegetation types characterize the study area: woodlands dominated by *Acacia drepanolobium*, the most common vegetation type in Laikipia [27]; savannas dominated by perennial grasses with widely spaced trees and shrubs; and bushlands with a discontinuous layer of perennial grasses and a broken canopy dominated by *Acacia mellifera*, *A*. *etbaica*, *A*. *brevispica* and *Grewia tenax* [24].

### Ethics statement

This study was reviewed and approved by the Office of the President of the Republic of Kenya and the Kenya Wildlife Service (permits KWS/BRM/5001, NCST/RRI/12/1/BS011, NCST/RRI/12/1/BS011/364 and NCST/RRI/12/1/BS011/18), and relevant research committees at University of California, Berkeley, University of California, Santa Cruz and Princeton University. The lion collaring protocol was reviewed and approved by the Animal Care and Use Committee, University of California, Berkeley, Protocol No. R191 (to LGF) and the Institutional Animal Care and Use Committee, University of California, Santa Cruz Permit No. WILMC1402 (to CW and TW). Lion anesthesia: lions darted with a combination of Medetomidine and Ketamine. The zebra collaring protocol was reviewed and approved by the Institutional Animal Care and Use Committee, Princeton University, Protocol No. 1845 (to DR). Zebra anesthesia: Kenya wildlife Service Veterinarians darted using M99. All species in this study are considered protected species under Kenyan Law. Population estimation of Grevy’s and Plains zebras was conducted using non-invasive line transect surveys and were conducted on private ranches.

### Lion monitoring

Lions are almost entirely nocturnal in Laikipia County and lion kills are rarely found due to dense bush. Formerly, tracking circling vultures was a reliable means of locating carcasses but the collapse of Kenya’s vulture populations due to predator poisoning [28, 16] reduced the reliability of this method. To find fresh kills, we relied on satellite telemetry. We captured female lions by calling them to a bait at night, immobilized them using tranquilizer darts with medetomidine and ketamine [29] (University of California, Berkeley, ACUC Protocol No. R191) and fitted them with GPS collars (Vectronic Aerospace, Berlin, Germany). The collars recorded locations every hour between 1800–0700 and sent data each morning via the Iridium satellite phone system. We displayed nightly movements in Google Earth and identified sites where the lions had been sedentary for at least two hours, defined as three or more hourly fixes within a 50 m radius. We visited these potential feeding sites within 48 h and searched for prey remains, identifying prey to species, sex, and age when possible. We developed prey profiles as percent of all kills by species. Some zebra remains (e.g. internal organs) could not be identified to species, and we allocated those to Grevy’s and Plain’s zebras according to the ratio of identified zebra kills. Lions and spotted hyenas (C*rocuta crocuta)* regularly appropriate kills from each other [30, 31], as well as from leopards and cheetahs, and it is rarely possible to determine definitively from remains and tracks which predator made the kill. We therefore assumed that all kills found at potential feeding sites were attributed to collared lions but recognize this potential source of error.

### Zebra monitoring

Zebras were darted from a vehicle using tranquilizer darts containing M99 (Princeton University IACUC Protocol No. 1835) under the supervision of a Kenya Wildlife Service veterinarian. Zebras were collared using custom-made GPS-GSM collars (Savannah Tracking Ltd. Nairobi, Kenya) and movement and ranging data were recorded at 15-minute intervals. Grevy’s zebra movements were monitored in 2007–2008 and Plain’s zebras were monitored in 2010–2011.

### Zebra population estimates

We used line transect surveys to estimate density and abundance of Plain’s and Grevy’s zebras. One hundred and seven 2-km transects and seventy-one 3-km transects were laid out systematically along existing roads in the southern and northern parts of the study area, respectively. Approximately 70 transects were located in bushland and the remaining surveys were in savannas and open woodland. It is usually inadvisable to use roads for line transect surveys [32, 33] if target species either use roads preferentially or avoid them entirely because roads may constitute unrepresentative habitat. This is not the case for our line transect surveys because: (1) animal trails traverse the habitat in a dense web so that roads do not represent special access for wildlife that is restricted elsewhere; (2) the land is privately owned and vehicle use of these roads is minimal, reducing the likelihood that animals will avoid the roads; (3) animals in this area are habituated to slow moving vehicles; and (4) upon examination of the distribution of detection distances, we did not find any evidence of heaping (attraction) or gaps (avoidance) in the vicinity of roads.

Southern transects were surveyed once in June 2014 and again in January 2015. Northern transects were surveyed twice during January 2015. All surveys were conducted in the morning between 0630 and 1000 and late afternoon between 1630 and 1830. Two observers, positioned on the roof of a vehicle at approximately 3 m height, recorded all individuals and groups of zebras encountered as part of a larger, multi-species survey. We measured observer-to-animal distances using laser rangefinders (Elite 1500, Bushnell Corp., Kansas City, MO, USA), and transect bearing and angle between animal and observer with digital compasses (High Gear Implus Footcare LLC, Orgeval, France). Perpendicular distance to the transect was calculated using the angle between transect and animal and radial distance from observer to animal.

We used DISTANCE 6.0 software [32] to analyse the combined surveys from the south and north parts of the study area. We analysed the data as exact perpendicular distances and truncated the Plain’s zebra dataset by deleting the largest 5% of perpendicular distances. We used the entire Grevy’s zebra dataset because of small sample sizes. We evaluated half normal, hazard, and uniform models with cosine and hermite polynomial adjustments and chose the final model based on a minimum Akaike information criterion [34] (Burnham and Anderson 2002).

### Modelling lion encounters with prey

A prey species is considered preferred when it is taken more than expected based on a random distribution of encounters conditioned on the abundance of predator and prey species [35, 36]. We used a random gas model [37] to determine the expected rate of encounters between collared lions and Plain’s and Grevy’s zebras. For a population of prey with density p_1_ moving at a velocity of v_1_ and predators with density p_2_ and velocity v_2_, the expected number of encounters that result in a predation attempt by a single predator unit, *E*_*pred*_, is a function of prey and predator densities, velocities, the distance D between predator and prey that defines an attempted predation encounter, and time t as follows:

$$E_{pred}=\frac{p_{1}}{p_{2}}\times \left[1-e^{-2\sqrt{v_{1}^{2}+v_{2}^{2}}p_{2}Dt}\right]$$ (1)

where density is expressed as individuals/km^2^, velocities are expressed as km/12 h, distance for an encounter is expressed as fraction of a km, and time is in days. The expected kill rate, E_kill_ given an encounter is simply E_pred_ times the proportion of attempted predation encounters that result in kills (S):

$$E_{kill}=E_{pred}\times S$$ (2)

To estimate E_pred_ and E_kill_, we used a bootstrap approach. Density of lions was estimated as the number of collared lions (n = 21) in a 2,105 km^2^ area defined by the union of all 95% minimum convex polygon home ranges for lions, or 0.01 lions/km^2^. We assumed that each collared lion was an independent hunting unit. Grevy’s and Plain’s zebra densities were estimated from line transect surveys as the cluster density since lions were expected to encounter a group of zebras and kill a single member of the group when an encounter resulted in successful predation. In the simulations, lion density was treated as a constant. Zebra densities were randomly chosen from normal distributions with observed density and standard deviations, truncated at 0.

To estimate velocities, we used hourly movements from satellite telemetry fixes (S1 Table, S1 Appendix, S2 Appendix) collected between 1800 and 0700 for 21 female lions (n = 101,419 hourly locations), 7 Plain’s zebra (n = 14,159 hourly locations) and 5 Grevy’s zebra (n = 16,297 hourly locations). We constructed velocities by randomly sampling 12 hourly movements and then summing. Because we use hourly straight line movement, our velocities are likely minimum estimates of potential movements over uneven terrain. To determine the maximum contact distance to initiate a predation attempt and the likelihood that an attack would result in a kill, we relied on data reported in the literature [30, 38, 39, 40, 41]. We selected 4 encounter distances (30 m, 40 m, 50 m, and 60 m) as the upper limits for encounter distances that would result in an attack. We estimated a constant time t as the mean time that a collared lion was tracked. Successful predation given an encounter has been measured within a range between 10–28% of attempts [30, 38, 39, 40, 42]. To estimate E_kill_, we used values of 10%, 15%, 20% and 25%. Each simulation was run 10,000 times and a mean, SD and 95% CI was calculated for each combination of parameters. We then compared the simulation kill rates over the course of the study to the estimated kill rates based on field data for the number of potential feeding sites, the proportion of actual feeding sites among potential feeding sites and the proportion of Plain’s and Grevy’s zebras among recovered kills.

## Results

### Lion and zebra movements

Between 27 November 2013 and 1 January 2015, we collared 21 female lions in 10 prides (S1 Table) and tracked their movements for an average of 226.5 days/female (SD = 132.3, range 43–515 days). Females moved on average 504 m/h (range of means: 380.7–668.5 m/h) and 6,575 m/12-h (range of means: 3,891–8,736 m). Between 19 November 2010 and 27 July 2011, we collared 6 Plain’s zebras and tracked their movements for an average of 184.3 days (range: 102–242 days). Plain’s zebras moved on average 314 m/h (range 187.3–379.0 m/h) and 4,125 m/12-hr (range 2,398–5,086 m). Between 13 June 2007 and 4 January 2008, we collared 5 Grevy’s zebra and followed their movements for an average of 111 days (range 38–206 days). Grevy’s zebras moved on average 335.2 m/h (range 238.6–426.1 m/h) and 3,781 m/12-hr (range 2,840–4,349 m).

### Prey densities

We conducted 387 line transect surveys totaling 904 km of survey effort, and observed 697 Plain’s zebra groups and 68 Grevy’s zebra groups (Table 1). Mean group size was 8.07 individuals (SD = 0.332) for Plain’s zebra and 2.38 individuals (SD = 0.348) for Grevy’s zebra. The best fitting DISTANCE model for Plain’s zebra was a hazard model with cosine adjustment terms and for Grevy’s Zebra the best fit was a hazard model with hermite adjustment term. We estimated the density of Plain’s zebra groups at 1.98/km^2^ (95% CI = 1.61–2.42) and individual density at 15.94/km^2^ (95% CI = 12.82–19.82). We estimated the density of Grevy’s zebra groups at 0.30/km^2^ (95% CI = 0.17–0.53) and individual density of 0.71/km^2^ (95% CI = 0.38–1.34). Coefficient of variation was higher for Grevy’s zebra compared to Plain’s zebra due to small sample sizes.

**Table 1:** DISTANCE parameter estimates. Distance parameter estimates with coefficient of variation (CV), degrees of freedom (DF) and 95% confidence interval (CI).

Species	Parameter	Estimate	CV	DF	95% CI
Plains Zebra	Encounter rate	0.771	9.65	386	(0.64, 0.93)
	Detection probability	0.560	3.72	695	(0.521, 0.602)
	Expected strip width	195.110	3.72	695	(181.37, 209.88)
	Cluster size	8.066	4.12	696	(7.44, 8.74)
	Cluster density	1.976	10.34	502.9	(1.62, 2.42)
	Individual density	15.941	11.13	662.89	(12.82, 19.82)
Grevy's Zebra	Encounter rate	0.075	15.36	386	(0.056, 0.102)
	Detection probability	0.208	25.04	66	(0.127, 0.341)
	Expected strip width	125.470	25.04	66	(76.69, 205.28)
	Cluster size	2.382	14.59	67	(1.78, 3.18)
	Cluster density	0.300	29.37	122.06	(0.17, 0.53)
	Individual density	0.714	32.79	170.76	(0.38, 1.342)

### Random gas models

We determined 3,993 instances during which a lion moved less than 50 m over 3 or more hourly fixes (potential feeding site) during 4,756 days of telemetry data. We investigated >3,000 potential lion feeding sites and located 768 kills (~25% of potential feeding sites: Table 2). Plain’s zebra were the top prey item (44.3% of kills: Table 2), followed by domestic cattle (*Bos Taurus*: 12.6% of kills). Only 2.0% of estimated kills were Grevy’s zebra. This represents an estimated 17.6 Plain’s zebra and 0.7 Grevy’s zebra killed per lion during an average 226.5 days that the 21 lions were followed, or 28.4 Plain’s zebra and 1.2 Grevy’s zebra killed per lion per year. The study area contains a full complement of predators, and lions are known to take prey from hyenas, leopards and cheetahs. Because our methods could not distinguish prey killed by lions from prey scavenged by lions, predation rates could potentially be lower.

**Table 2:** Distribution of prey species among 768 presumptive collared lion kills.

Species	count	%	Cumulative %	rank
**Plains Zebra**	340	44.3%	44.3%	1
Cow	97	12.6%	56.9%	2
Eland	65	8.5%	65.4%	3
Reticulated Giraffe	40	5.2%	70.6%	4
Unknown	36	4.7%	75.3%	5
Common Warthog	29	3.8%	79.0%	6
Impala	26	3.4%	82.4%	7
African Buffalo	24	3.1%	85.5%	8
Beisa Oryx	23	3.0%	88.5%	9
Jackson's Hartebeest	16	2.1%	90.6%	10
**Grevy's Zebra**	15	2.0%	92.6%	11
Grant/Thomson Gazelle	12	1.6%	94.1%	18
Sheep/Goat	9	1.2%	95.3%	12
Camel	7	0.9%	96.2%	13
Ostrich	6	0.8%	97.0%	19
Aardvark	4	0.5%	97.5%	15
Donkey	4	0.5%	98.0%	16
African Elephant	4	0.5%	98.6%	17
Water Buck	4	0.5%	99.1%	20
Gerenuk	2	0.3%	99.3%	22
Cape Hare	1	0.1%	99.5%	23
Hippopotomus	1	0.1%	99.6%	24
Horse	1	0.1%	99.7%	25
Greater Kudu	1	0.1%	99.9%	26
Vulturine Guinea Fowl	1	0.1%	100.0%	27

**Fig 3:**
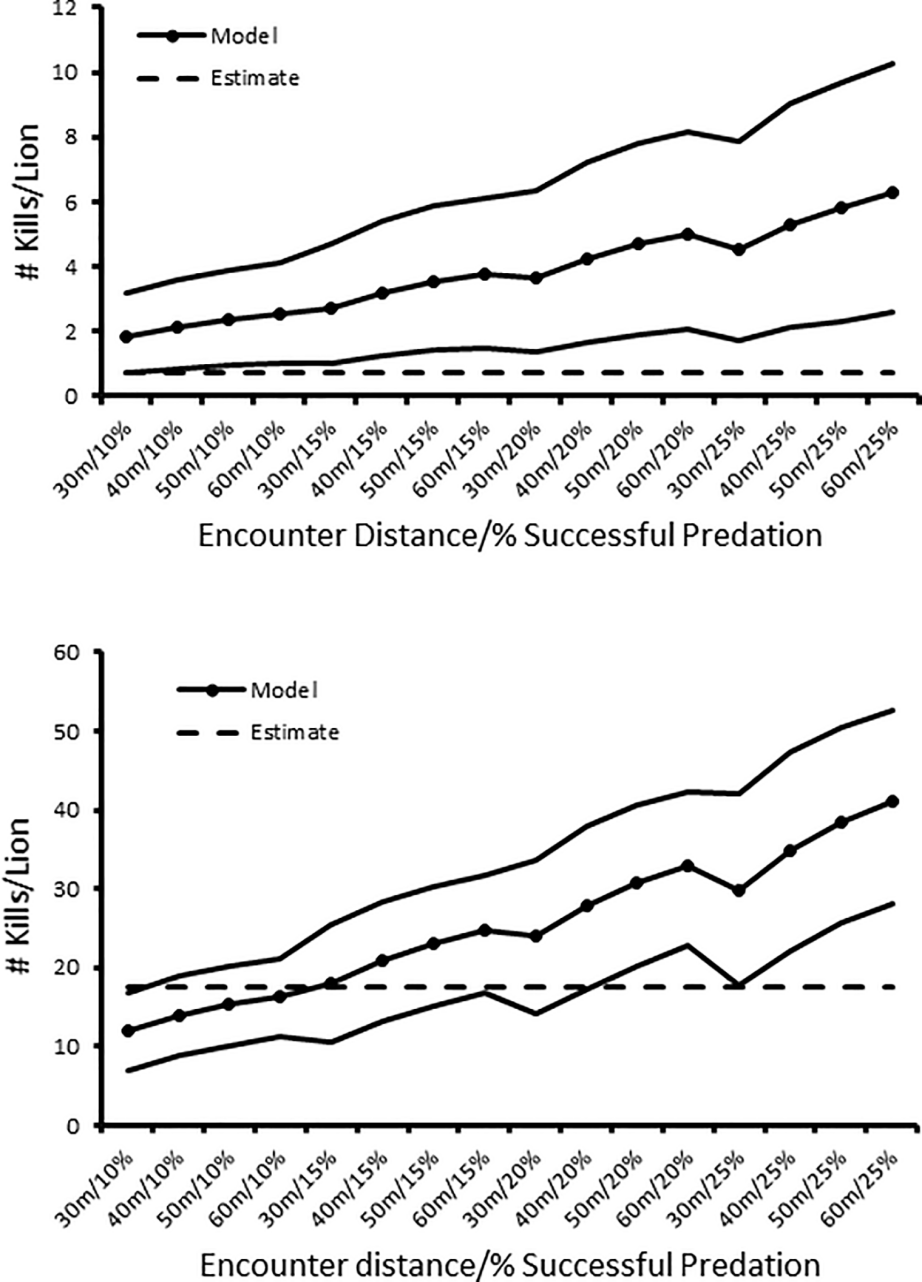
Random gas model results. Expected predation rates during the course of the study based on field estimates (dashed line) and random encounter models (points plus 95%CI lines) using different scenarios of distance at which an encounter is initiated and successful predation rates for (a.) Plain’s zebra kills/female lion and (b.) Grevy’s zebra kills/female lion.

Our field-based estimates of predation rates on Plain’s zebra were similar to those generated using the random gas model. Under the most conservative scenario (encounter distance 30 m, predation success rate 10%), the modeled estimate of predation was significantly lower than field-based estimate of predation (Fig 3A). For nine other conservative and moderate scenarios of encounter distance and successful predation rates (40 m/10% to 40 m/20%; Fig 2A), modeled estimates were not significantly different from the field-based estimate. Under the six most liberal scenarios (50 m/20% to 60 m/25%; Fig 2A) modeled estimates were significantly higher than the field-based estimates.

Expected predation rates by lions on Grevy’s zebra generated using the random gas model were significantly higher than the field-based estimates for all but the most conservative scenario (encounter distance 30 m, success rate 10%: Fig 2B).

## Discussion

Our results indicate that the observed lion predation on Grevy’s zebra is less than expected due to chance alone in 15 of 16 (94%) of cases. In comparison, predation on Plain’s zebras is as expected (9 scenarios: 56%) or less than what would be expected due to chance alone (6 scenarios: 38%). Observed predation on Grevy’s zebra was consistently less than expected for all model scenarios, equaling the lower limit of the confidence interval only for the most conservative scenario (30 m encounter distance, 10% successful predation). As modelled encounter distance and success rate increased, the disparity between observed and expected predation rates also increased, with the random gas models predicting significantly more predation than observed in the field for both subspecies. Because predicted selective predation by lions on Grevy’s should show a greater rate of encounters and therefore a larger number of observed kills compared to random encounters, our data indicate that lions are not preying selectively on Grevy’s zebras in Laikipia County.

Our results support the hypothesis of Georgiadis et al. [23] that lions do not regulate Plain’s zebra densities in Laikipia County. Only under the most conservative model scenario (30 m encounter distance, 10% predation success) did the estimated predation rate exceed the modeled predation rate. In all other scenarios, the estimated predation rate was similar or significantly lower than the modeled predation rates.

### Use of the random gas model

The random gas model has been applied widely as a null hypothesis to model encounter rates within and among species [37, 43, 44]. Under the random gas model, encounter rates are a function of encounter distances, rates of movements of predators and prey, and density of predators and prey. Model assumptions include: (1) individuals are randomly distributed; (2) each individual’s movements are independent, straight line and equally likely in all directions and; (3) velocities are normally distributed. Despite the obvious fact that animals do not behave like gas particles, the model has proven useful as a model of animal movements in a wide variety of situations, including density estimation [44, 45], intergroup encounters [46, 47, 48], and predator-prey encounters [43, 49, 50]. Hutchison and Waser [37] demonstrated that violation of the random distribution assumption does not bias expected encounter rates. In this study, violations of the assumptions 2 (movement trajectories) and 3 (velocity) tend to result in underestimation of expected encounter rates making our simulations of encounter rates conservative. Our inability to unambiguously distinguish lion predation from kleptoparasitism may have inflated our estimate of actual lion predation rates, also making the comparison of estimated and simulated predation rates conservative.

Rubenstein [25] modeled population trajectories for Grevy’s zebras using a two-sex age structured stochastic population model [51] with effects of density dependence and rainfall to estimate the population trajectory over 30 years. He demonstrated that a population of 150 Grevy’s with survival rates characteristic of the Laikipia Grevy’s zebra population might decline by 15% over 30 years. Rubenstein’s models indicated that when the percentage of juveniles and foals in the population reached 30%, the population would stabilize. Rubenstein et al. [14] report a steady improvement in proportion of juveniles and foals from 9% in 2004 to 28% in 2016, suggesting that the population is stabilizing.

Threats to Grevy’s zebras [14] include: 1) habitat degradation due to overgrazing by livestock; 2) competition with livestock over access to water and food; 3) disease from contact with unvaccinated livestock; 4) local hunting; 5) predation; and 6) development corridors. Based on our observations and models, we rank habitat degradation and competition with livestock and Plain’s zebras as the most important threats to Grevy’s zebra recovery. Between 1997 and 2012, the ratio of livestock to Grevy’s zebra in Laikipia doubled from 483 to 864 individual livestock per Grevy’s zebra [22]. Because livestock can monopolize the limited access points to water and unvaccinated livestock carry transmittable disease, the increase in occurrence of domesticated ungulates appears to represent a significant threat. Plains zebra greatly outnumber Grevy’s zebra (22 Plains zebra for each Grevy’s zebra) and Grevy’s zebras feed less in the presence of Plain’s zebras [25]. The combination of displacement and threats due to livestock, and the competitive dominance of Plain’s zebras may ultimately limit the recovery of Grevy’s zebras. In contrast, predation by lions, does not appear to represent a major limiting factor to population growth, especially when compared to other potential threats.

## Supporting information

S1 Table Details of lion collar operation. Dates that collars were operational, number of sample days, hourly distance traveled (SD), and daily distance traveled (SD) for female lions, Plains zebra and Grevy's zebra. Lions with numbers were recaptured more than once and collar either changed or repaired during study.
(DOCX)

S1 Appendix Hourly movement data. Hourly movement data for African lions, Grevy’s zebras and Plains zebras used in simulations of encounter rates.
(XLSX)

S2 Appendix Comparison of ecological conditions. The compatibility of temporally disconnected movement data.
(DOCX)
