# Supplementary material for: Resolving a conservation dilemma: Vulnerable lions eating endangered zebras
Source: PLoS One. 2018 Aug 29;13(8):e0201983. doi: 10.1371/journal.pone.0201983 (PMC6114509; doi:10.1371/journal.pone.0201983)
Supplement: S1 Table — Dates that collars were operational, number of sample days, hourly distance traveled (SD), and daily distance traveled (SD) for female lions, Plains zebra and Grevy's zebra. Lions with numbers were recaptured more than once and collar either changed or repaired during study. (DOCX) [file pone.0201983.s001.docx]

| Table S1. Dates that collars were operational, number of sample days, hourly distance traveled (SD), and daily distance traveled (SD) for female lions, Plains zebra and Grevy's zebra. Lions with numbers were recaptured more than once and collar either changed or repaired during study. | | | | | | | |
| --- | --- | --- | --- | --- | --- | --- | --- |
| Name | Collar Number | Capture Date | Data last signal | Age | Number 12-H Days | Mean distance hourly (m) | Mean daily distance (m) |
| Apethe | 16660 | 07/25/15 | 12/31/15 | Adult | 160 | 618 (425) | 7899 (5459) |
| Astrid 1 | 16660 | 11/13/14 | 03/12/15 | Adult |  |  |  |
| Astrid 2 | 16666 | 03/12/15 | 12/31/15 | Adult | 413 | 381 (290) | 4933 (3747) |
| Bella | 17090 | 04/20/15 | 08/28/15 | Old Adult | 142 | 598 (522) | 7876 (4823) |
| Boadicea | 13817 | 07/27/15 | 12/31/15 | Adult | 158 | 507 (357) | 6594 (4491) |
| Caelem | 16664 | 10/19/14 | 02/21/15 | Adult | 126 | 490 (290) | 6497 (3771) |
| Cecia | 16666 | 09/23/14 | 01/28/15 | Adult | 127 | 606 (394) | 7933 (5155) |
| Charlotte | 17083 | 11/19/15 | 12/31/15 | Adult | 43 | 398 (325) | 5257 (4247) |
| Chessie 1 | 13813 | 12/06/13 | 03/14/15 | Adult |  |  |  |
| Chessie 2 | 16661 | 06/01/15 | 11/11/15 | Adult |  |  |  |
| Chessie 3 | 17087 | 03/31/15 | 05/17/15 | Adult | 389 | 486 (394) | 6190 (4785) |
| Cindy | 13803 | 11/27/13 | 12/23/14 | Adult | 376 | 390 (278) | 5054 (3614) |
| Diabla | 16663 | 04/02/15 | 12/31/15 | Young Adult | 274 | 560 (377) | 7217 (4826) |
| Eloise 1 | 16665 | 11/09/14 | 11/13/14 | Young Adult |  |  |  |
| Eloise 2 | 16665 | 11/25/14 | 03/31/15 | Young Adult | 130 | 429 (232) | 5648 (3044) |
| Farasi | 16667 | 09/24/14 | 01/24/15 | Adult | 122 | 615 (437) | 8069 (5775) |
| Kasi | 13816 | 05/29/14 | 07/22/14 | Adult | 54 | 441 (255) | 5715 (3328) |
| Lisa | 13817 | 05/22/14 | 03/18/15 | Adult | 300 | 577 (351) | 7962 (4594) |
| Maggie | 16667 | 05/09/15 | 12/31/15 | Adult | 237 | 479 (304) | 6179 (3875) |
| Margarita | 16663 | 10/01/14 | 01/26/15 | Adult | 117 | 593 (304) | 7719 (3947) |
| Merimela 1 | 13816 | 09/13/14 | 05/01/15 | Adult |  |  |  |
| Merimela 2 | 17088 | 07/03/15 | 12/31/15 | Adult | 288 | 506 (302) | 6574 (3907) |
| Mulandama | 13815 | 02/16/14 | 07/18/15 | Adult | 515 | 522 (348) | 6680 (4461) |
| Shandy | 16661 | 09/26/14 | 01/24/15 | Adult | 120 | 669 (456) | 8736 (5937) |
| Stella 1 | 13813 | 03/30/14 | 11/28/14 | Old Adult |  |  |  |
| Stella 2 | 13814 | 12/12/14 | 02/09/15 | Old Adult | 277 | 421 (313) | 5479 (4040) |
| Victoria 1 | 16659 | 10/15/14 | 02/16/15 | Old Adult |  |  |  |
| Victoria 2 | 16659 | 04/13/15 | 12/31/15 | Old Adult | 388 | 300 (273) | 3891 (3543) |
|  |  |  |  |  |  |  |  |
| Plains Zebra-1 | pz_1072 | 11/19/10 | 07/27/11 | Adult | 242 | 356 (445) | 4501 (2113) |
| Plains Zebra-2 | pz_1078 | 11/19/10 | 07/15/11 | Adult | 238 | 334 (578) | 5086 (2344) |
| Plains Zebra-3 | pz_1080 | 11/23/10 | 07/07/11 | Adult | 102 | 321 (424) | 4011 (2468) |
| Plains Zebra-4 | pz_1082 | 11/22/10 | 07/01/11 | Adult | 131 | 379 (463) | 4811 (2188) |
| Plains Zebra-5 | pz_1083 | 11/23/10 | 07/01/11 | Adult | 205 | 306 (457) | 3941 (2394) |
| Plains Zebra-6 | pz_1084 | 11/23/10 | 05/30/11 | Adult | 188 | 187 (377) | 2398 (2734) |
|  |  |  |  |  |  |  |  |
| Grevy's Zebra-1 | gz_1 | 06/13/07 | 09/09/07 | Adult | 79 | 426 (794) | 4140 (2659) |
| Grevy's Zebra-2 | gz_2 | 06/13/07 | 01/04/08 | Adult | 206 | 359 (493) | 4113 (2795) |
| Grevy's Zebra-3 | gz_3 | 06/14/07 | 09/19/07 | Adult | 98 | 363 (446) | 4349 (1797) |
| Grevy's Zebra-4 | gz_6 | 06/16/07 | 07/23/07 | Adult | 38 | 290 (392) | 3462 (1492) |
| Grevy's Zebra-5 | gz_7 | 06/16/07 | 10/27/07 | Adult | 134 | 239 (425) | 2840 (2782) |
|  |  |  |  |  |  |  |  |
