# Supplementary material for: Resolving a conservation dilemma: Vulnerable lions eating endangered zebras
Source: PLoS One. 2018 Aug 29;13(8):e0201983. doi: 10.1371/journal.pone.0201983 (PMC6114509; doi:10.1371/journal.pone.0201983)
Supplement: S2 Appendix — The compatibility of temporally disconnected movement data. (DOCX) [file pone.0201983.s003.docx]

**S2 Appendix: The compatibility of temporally disconnected movement data**

The satellite telemetry data used to calculate hourly movements in this study were collected over 3 non-overlapping time periods in 2007-2008, 2010-2011 and 2013-2015 for Grevy’s zebra, Plains zebra and African lion, respectively. Laikipia County has a dynamic climate and is subject to alternating periodic droughts and heavy rains (Franz et al. 2010). To assess whether the ecological conditions were similar during the three time periods, we reviewed the literature on zebra movements, evaluated abundance and density of zebras at two scales – extensive aerial surveys and intensive line transect surveys – and evaluated the impact of rainfall on herbaceous vegetation.

**Rainfall, Temperature and herbaceous vegetation:** Zebras rely on herbaceous vegetation for food and the abundance of herbaceous vegetation is affected by climate. Mean monthly minimum and maximum temperatures varied by 1 ^o^C or less and mean monthly rainfall was similar across the three study periods (Mpala Research Centre unpublished data; Table 1). Young et al. (2013) showed that rainfall was one of the most important factors affecting vegetation height and cover, two important determinants of vegetation biomass. Because rainfall was similar across the three study periods, we assume that herbaceous vegetation biomass was comparable among the three study periods.

| Table 1. Zebra abundance from aerial surveys, zebra density from line transects, and climate data for three telemetry sampling periods and mean values for the 2007-2015 time period. | | | | | | |
| --- | --- | --- | --- | --- | --- | --- |
|  | Sampling period | | |  | 2007/2015 | |
| Metric | 2007/08 | 2010/11 | 2013/15 |  | Mean | SD |
| Grevy's zebra density LT (/km^2^) | 0.98 | 0.91 | 0.71 |  | 0.83 | 0.38 |
| Grevy's zebra abundance | 419 | 425 | 435 |  | 425 | 123 |
| Plains zebra density LT (/km^2^) | 7.5 | 12.3 | 15.9 |  | 13.8 | 8.03 |
| Plains zebra abundance | 19350 | 10689 | 17209 |  | 15,457 | 4,842 |
| Monthly Rainfall (mm) | 45.3 | 32.6 | 41.9 |  | 53.6 | 49.66 |
| Min Temp (^O^C) | 14.5 | 14.0 | 13.5 |  | 13.4 | 2.27 |
| Max Temp (^O^C) | 28.3 | 28.7 | 28.8 |  | 28.3 | 1.53 |

**Hourly Movements:** We found only two studies on Grevy’s zebra movements. Hostens (2009) analyzed telemetry movement data for 16 Grevy’s zebras in Samburu County (North of Laikipia County) between June 2006 and August 2008. Average hourly movement of Grevy’s zebras was estimated at 0.42 km/h. Wheeler (2013) followed 10 Grevy’s zebras in Samburu County, Kenya (~70 km northeast of Mpala Ranch) between 0ctober 2010 and December 2012. Wheeler reported an average hourly movement of 0.4 km/h. Finally, Kartzinel et al. (2015) followed 4 grevy’s zebra in 2014 with an average hourly movement of 0.48 km/h. These hourly movements are consistent with our estimate of 0.34 km/h and indicate that Grevy’s zebra movements are relatively constant in space and time.

Plains zebras are more variable in their hourly movements depending on context. Fischhoff et al (2007) followed four Plains zebras in a 394 km^2^ fenced conservancy in Laikipia County for 39 days between July 2003 and August 2005. Zebras averaged 0.54 km/h at night in woodland habitat and 0.77 km/h in grassland habitat. We note that lion density was relatively high at 8 lions per 100 km^2^ and the authors attributed the rapid movements in grasslands at night to predation threat. Lion density was 5.3/100 km^2^ in our study area and 87.4% of Plains zebra movements were less than 0.77 km/h. Two Plains zebra followed in Laikipia county in April-May 2014 averaged 0.42 km/h (Kartzinel et al. 2015). We assume that predation risk for zebras at night in our study area was lower and this may account for slower hourly movements at night.

Two additional studies in Botswana examined directed movements in Plains zebras. Brooks and Harris (2007) examined directed movements of plains zebras up to 3.7 km from spatially restricted water holes and foraging patches. Average hourly movements were reported for only part of the day spent foraging and averaged 1.13 km/h. Bartlam-Brooks et al. (2011) report on movements of plains zebras during migrations from July 2007 to November 2009. Of 16 zebras, hourly movements averaged 1.05 km/h. In our study, 91.8% of Plains zebra movements were less than 1 km/h.

**Density and Abundance:** We used aerial surveys conducted in February-March 2001,2003, 2004, 2005, 2006 2008 2010 2012 and 2016 to estimate minimum abundance of Plains and Grevy’s zebras in Laikipia County (extensive surveys: Georgiadis et al 2007, Kinnaird et al. 2012). We also used line transect surveys to estimate the density of Plains and Grevy’s zebras across 200-400 km^2^ of Laikipia in the months of January and June between 2008 and 2015 (intensive surveys: unpublished data), and additional surveys in February 2015. Aerial surveys conducted between 2001 and 2016 (Kinnaird et al 2012, unpublished data) and restricted to the Wildlife Friendly ranches where our study was centered, indicate that Plains zebra have declined slightly over time (1.05%/yr) while Grevy’s zebras have been declining at a higher rate (4.22%/yr). However, aerial censuses conducted around the time of our telemetry studies indicate that the Grevy’s zebra population in our study area was similar in all three survey periods (Table 1). Grevy’s Zebra densities estimated from line transect surveys, show a decline between 2010/2011 and 2013/2015 but all estimates were within one standard deviation of the mean. Aerial surveys of Plains zebra show that the population declined between 2007/08 and 2010/11, but then recovered between 2010/11 and 2013/15. All three estimates are within 1 standard deviation of the 2007-2015 average. In conclusion, we believe that environmental conditions were similar during sampling periods and that zebra populations varied but were not significantly different during the three sampling periods.

The literature indicates that Grevy’s zebra move at the similar rates across studies conducted in different locations and over different time periods. Regarding hourly movements of Plains zebras, we cannot draw firm conclusions from the few studies available; most studies involved a relatively high predation threat (compared to our study), directed movements and migration. A small sample of movements from Laikipia in 2014 was similar to our results. We assume that Plains Zebra movements are driven by water dependence and availability of forage rather than by differences in density (Rubenstein 2010), and therefore we assume that an hourly movement rate of 0.31 km/hr can be applied to Plains zebra in our study site.

**REFERENCES**

Bartlam-Brooks, H.L.A, M.C. Bonyongo and S. Harris. 2011. Will reconnecting ecosystems allow long-distance mammal migrations to resume? A case study of a zebra *Equus burchelli* migration in Botswana. Oryx 45:210-216.

Brooks, C.J. and S. Harris. 2008 Directed movement and orientation across a large natural landscape by zebras, *Equus burchelli antiquorum*. Animal Behaviour 76:277-285.

Fischhoff, I.R., S.R. Sundaresan, J. Cordingley and D.I. Rubenstein. 2007. Habitat use and movements of plains zebra (Equus burchelli) in response to predation danger from lions. Behavioural Ecology 18:725-729.

Franz, T. E., K. K. Caylor, J. M. Nordbotten, I. Rodrı´guez-Iturbe, and M. A. Celia. 2010. An ecohydrological approach to predicting regional woody species distribution patterns in

dryland ecosystems. Advances in Water Resources 33:215–230.

Hostens, E. 2009. Modelling the migration of Grevy’s zebra in function of habitat type using remote sensing. Unpublished MSc. Thesis, University of Gent. 126 pp.

Georgiadis N.J., J.G.N. Olwero, G. Ojwang G, and S.S. Romanach. 2007. Savanna herbivore dynamics in a livestock-dominated landscape: I. Dependence on land use, rainfall, density and time. Biological Conservation. 137:46-472.

Kartzinel, T., P. Chen, T. Coverdale, D. Erickson, J. Kress, D.I. Rubenstein, W. Wang and R. Pringle. 2015. DNA metabarcoding illuinates dietary niche partitioning by large African herbivores. Proceedings of the National Academy of Sciences. 112:8019-8024.

Kinnaird M., T. O’Brien and G. Ojwang. 2012. Sample Count Aerial Surveys as a Monitoring Tool for Wildlife and Livestock: A Case Study from Laikipia County. Report to the Laikipia Wildlife Forum, Nanyuki, Kenya. 22 pp.

Rubenstein D.I. 2010. Ecology, social behavior and conservation in zebras. Advances in the Study of Behavior. 42:231-258.

Wheeler, L.L. 2013. Using telemetry data to study the behavioural responses of Grevy’s zebra in a pastoral landscape in Samburu, Kenya. Unpublished MSc. Imperial College. 45 pp.

Young, H.S., D.J McCauley, K.M. Helgen, J.R. Goheen, E. Otarola-Castillo, T.M. Palmer, R.M. Pringle, T.P. Young, and R. Dirzo. 2013. Effects of mammalian herbivore declines on pplant communities: observations and experiments in an African savanna. Junrnal of Ecology 101:1030-1041.
